# Mechanism of Interfacial Slippage in the Micro-Triangle and Composite Fiber Membrane Characteristics in Rotary-Force Spinning

**DOI:** 10.3390/polym17233235

**Published:** 2025-12-04

**Authors:** Jianwei Ma, Meng Zhang, Shuo Zhao, Zhiming Zhang, Zhen Chen, Qiaoling Ji

**Affiliations:** 1 Hubei Digital Textile Equipment Key Laboratory, Wuhan Textile University, Wuhan 430200, China; 2315373070@wtu.edu.cn (J.M.); 2415033009@wtu.edu.cn (M.Z.); 2415373063@wtu.edu.cn (S.Z.); czhen@wtu.edu.cn (Z.C.); stasy00@126.com (Q.J.); 2 School of Mechanical Engineering and Automation, Wuhan Textile University, Wuhan 430200, China; 3 Zhejiang Provincial Innovation Center of Advanced Textile Technology, Shaoxing 312000, China

**Keywords:** rotational-force spinning, micro-triangle, liquid–liquid slip, composite fiber membrane, simulation analysis

## Abstract

Composite fiber membranes fabricated via rotational-force spinning have become widely applied in biomedicine, energy, and environmental fields owing to their excellent properties. Improving their functional performance and fabrication quality has therefore become a key research focus. Rotational-force spinning is a simple and efficient technique in which high-speed motor rotation ejects polymer solutions from a nozzle to form fibers. However, the influence of polymer flow behavior within the nozzle on fiber formation remains insufficiently understood. In this study, the flow characteristics within the micro-triangle and the liquid–liquid slip phenomenon were investigated using a core–shell spinning device. Numerical simulations were conducted to analyze velocity differences between two polymer solutions under varying motor speeds and polyoxyethylene (PEO) concentrations. The results demonstrate that increasing PEO concentration and motor speed decreases slip velocity, thereby stabilizing the flow. Complementary experiments were performed using PEO and hydroxyethyl cellulose (HEC) solutions under controlled conditions. Mechanical testing, scanning electron microscopy (SEM), and thermogravimetric analysis (TG) were employed to assess the mechanical performance, thermal stability, morphology, and fiber diameter distribution of the composite membranes. Overall, the findings highlight the critical role of liquid–liquid slip in fiber formation and provide valuable insights for the controlled fabrication of high-quality composite fibers, offering a foundation for future research.

## 1. Introduction

Composite fibers are formed by combining two or more materials with distinct properties through specific processing techniques, enabling performance that a single fiber alone cannot achieve [1]. Owing to their versatility, structural tunability, and cost-effectiveness, composite fiber membranes have received increasing attention and have been widely applied in medicine, environmental protection, textiles, bioengineering, and energy [2,3,4,5,6]. Furthermore, by adjusting the internal structure of the tank in rotary-force spinning devices, composite fibers with side-by-side, core–shell, or island-in-the-sea structures can be fabricated [7,8,9].

In the early stages of composite fiber development, different fiber types were simply mixed and spun into yarns. With advancements in science and technology, preparation methods have progressively evolved. At present, the most commonly employed techniques include melt-blown spinning, wet spinning, electrospinning, and rotary-force spinning [10,11,12,13]. Melt-blown spinning is an advanced technique for fabricating ultrafine fibers. In this process, two thermoplastic polymers are separately melted and co-extruded through a spinneret to form fine molten streams, which are then stretched by high-speed hot airflow and solidified under cooling air. This method enables large-scale production with high efficiency, but involves complex process control and material limitations [14]. Electrospinning employs a high-voltage electrostatic field to stretch polymer solutions or melts polymers into ultrafine fibers. While capable of producing nanoscale fibers with unique structures, it suffers from low production efficiency, difficulties in scale-up, reliance on high voltages, and environmental concerns [15].

**Figure 1:**
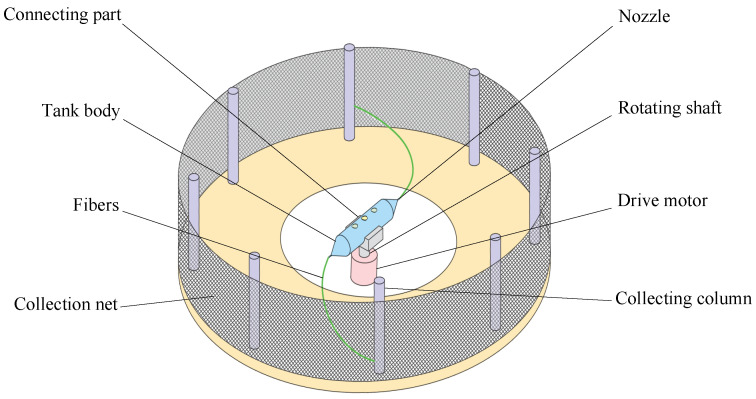
Structure diagram of rotary-force spinning device.

Rotary-force spinning is a technique for producing composite fibers, utilizing centrifugal force generated by a motor to rotate a tank at high speed, thereby ejecting the polymer solution through the nozzle. Compared to melt-blown spinning and electrospinning technologies, rotary-force spinning presents several advantages, including enhanced production efficiency, simplified operation, and compatibility with various thermoplastic polymer solutions or melts. As such, this technology holds significant potential for applications in biomedical and functional materials fields, including tissue engineering scaffolds, drug delivery systems, and smart textiles [16]. As illustrated in Figure 1, the rotary-force spinning device consists of a drive motor, a rotating shaft, a tank body, a connector, and multiple collecting columns. Existing studies indicate that the shape and quality of composite fibers are directly influenced by motor speed, polymer solution concentration, and nozzle diameter [17,18,19].

Composite fibers produced via rotary-force spinning exhibit excellent properties. Li et al. fabricated TiO_2_/PVDF/CA composite micro-nano fibers using rotary-force spinning. These fibers exhibited effective degradation of various dye concentrations and demonstrated good recyclability [20]. Morales et al. employed rotary-force spinning to fabricate a composite material consisting of poly(methyl methacrylate) and TiO_2_ nanoparticles (PMMA/NPsTiO_2_) with distinct morphologies: spherical (NPs0D) and one-dimensional (NPs1D). Various analytical techniques were used to characterize the obtained composites, revealing that PMMA/NPs0D and PMMA/NPs1D achieved MB photocatalytic degradation rates of 28.8% and 18.3%, respectively [21]. Zhang et al. fabricated water-soluble polyvinyl alcohol/carboxymethyl chitosan (PVA/CMCS) blended fiber films via a planar collection rotary-force spinning device. The results indicated that the maximum tensile strength and elongation at break of the PVA/CMCS blended fiber film reached 3.28 MPa and 29.52%, respectively. The PVA16/CMCS2 blend exhibited antimicrobial rates of 72.05% and 21.36% against Staphylococcus aureus and Escherichia coli, respectively [22]. Mary et al. developed a fiber pad by incorporating a bioactive cassia seed (CA) extract into polycaprolactone (PCL) via rotary-force spinning. SEM and gas chromatography-mass spectrometry (GC-MS) analyses confirmed that CA-PCL nanofiber pads are suitable as tissue engineering scaffolds for wound healing applications [23]. 

**Figure 2:**
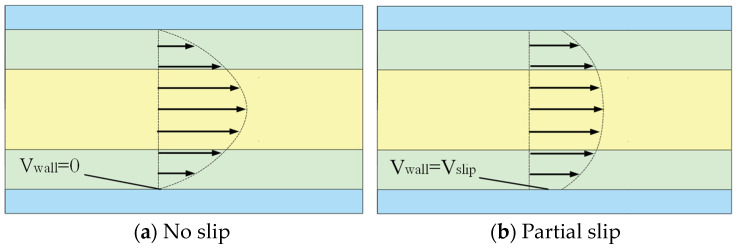
Fluid boundary condition.

At the beginning of the formation of the basic theory of fluid mechanics, most researchers assumed that the fluid velocity at a solid surface was zero, establishing the “no-slip condition.” However, advancements in experimental techniques and the accumulation of extensive experimental data led researchers to question the universality of the no-slip hypothesis. Eventually, they discovered the existence of relative slip between the moving fluid and the solid wall, as illustrated in Figure 2. Today, research on the slip phenomenon has become a critical area in fluid mechanics [24,25,26,27]. Poesio et al. demonstrated that fluid density distribution governs apparent slip at liquid–liquid interfaces, which is independent of shear rate and solely determined by intermolecular interactions [28]. Ye et al. found that in rotary-force spinning, polymer solution wall slip within the nozzle contributes to an increase in the average composite fiber diameter. Liquid–liquid slip influences both the surface morphology and internal structure of composite fibers [29].

During rotary-force spinning, the two polymer solutions in the tank rapidly move toward the nozzle due to the high-speed rotation induced by the motor. At this stage, the polymer solution experiences viscous and centrifugal forces, forming a conical droplet at the nozzle exit, termed the “micro-triangle”. “The micro-triangle resembles the “Taylor cone,” which forms during electrospinning when a solution in an injection needle is stretched by a strong electric field. Currently, research on the micro-triangle remains limited. Analyzing prior studies on the Taylor cone phenomenon in electrospinning could advance research on the micro-triangle, thereby enhancing the quality of spun composite fibers. Hao et al. regulated the yield, morphology, and internal structure of nanofibers produced via high-speed electrospinning by optimizing the Taylor cone [30]. Singh et al. discovered that adjusting the feed rate and applied voltage during electrospinning proportionally alters the Taylor cone height relative to the nanofiber diameter [31].

In this study, a core–shell rotational-force spinning device was employed to investigate the liquid–liquid slip phenomenon occurring in the micro-triangle during composite fiber fabrication. The formation mechanism of the micro-triangle flow and the interfacial slip between the two polymer solutions were analyzed through parametric modeling. The focus of this study is to elucidate the unique mechanism by which the slip phenomenon in a high-speed centrifugal field influences the fiber-forming process. Although the physical mechanisms by which interfacial slip affects flow and mixing have been widely studied in microfluidics, how interfacial slip regulates interfacial dynamics and subsequently influences solid-fiber structure formation in the high-intensity shear field of rotational spinning remains insufficiently understood [32,33]. To address this gap, numerical simulations were conducted to obtain the flow-field patterns and velocity distributions within the tank and nozzle, and the effects of key process parameters on slip behavior were systematically examined. Based on spinning experiments, this work analyzes how motor speed and polymer-solution concentration regulate the structure and properties of composite fiber membranes by altering the slip state, thereby clarifying the intrinsic relationships among slip behavior, interfacial mixing, fiber microstructure, and macroscopic properties.

## 2. The Mechanism of Micro-Triangle Slip in Rotational-Force Spinning

### 2.1. Formation Model of Micro-Triangle

During composite fiber formation via rotational-force spinning, the two polymer solutions are stored separately in the inner and outer tanks and transported to the nozzle outlet under centrifugal force. As the solutions flow through the inner and outer nozzles, liquid–wall slip first occurs along the inner nozzle wall, followed by liquid–liquid slip within the inner nozzle. With increasing motor speed, the polymer solution in the micro-triangle at the nozzle outlet forms a conical compound droplet under the combined effects of centrifugal force and static pressure, leading to expansion. Subsequently, air resistance induces droplet stretching and necking, resulting in the formation of a composite jet.

### 2.2. Micro-Triangle Liquid–Liquid Slip Model

**Figure 3:**
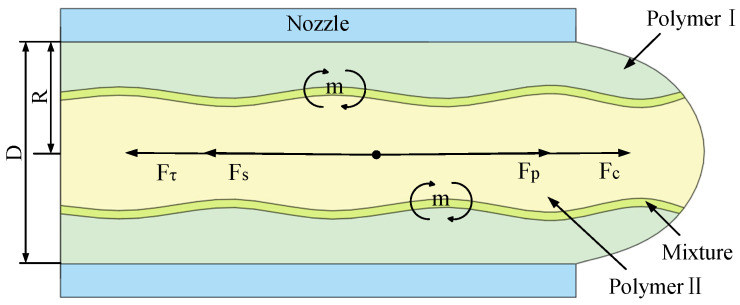
Force diagram of the micro-triangle polymer solution at the nozzle.

As illustrated in Figure 3, during rotational-force spinning, two polymer solutions form a micro-triangle at the nozzle. Assuming the two polymer solutions behave as an incompressible fluid, they are primarily influenced by static pressure (*F_p_*), centrifugal force (*F_c_*), surface tension (*F_s_*), and viscous force (*F_τ_*) within the nozzle. Among these forces, static pressure and centrifugal force drive the polymer solution toward the nozzle exit, whereas surface tension and viscous force resist its flow. By analyzing the axial forces acting on the micro-triangle polymer solution, the force equation is derived as follows:

(1) $$\sum_{i=1}^{n}F_{pi}+\sum_{i=1}^{n}F_{ci}\cdot ey=\sum_{i=1}^{n}\left(F_{si}+F_{\tau i}\right)\cdot ey$$

**Figure 4:**
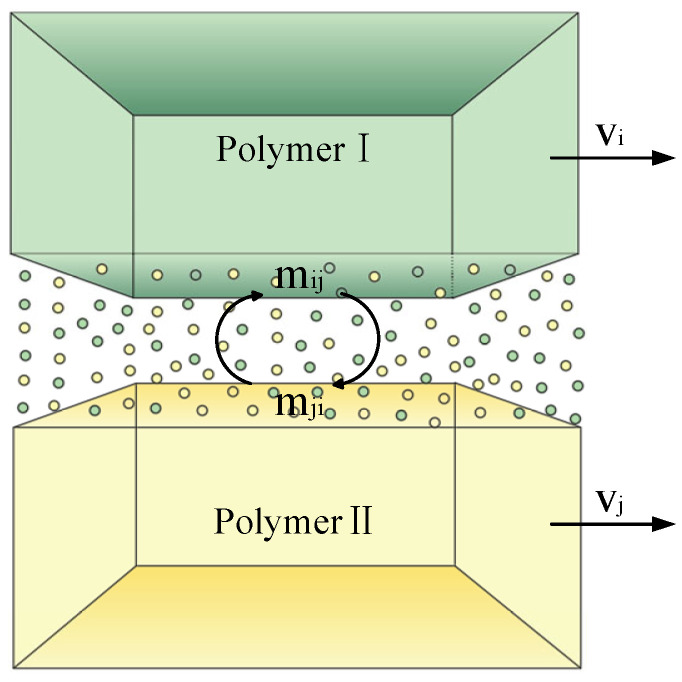
Molecular diffusion diagram of a mixing region.

When the tank rotates at high speed, both polymer solutions behave as continuous media. Due to differences in their physical properties, and under the influence of multi-field coupling effects, interfacial slip occurs between the two solutions, resulting in a two-phase flow. During this process, an interface is formed where the two polymer solutions come into contact. Across this interface, molecular coupling and mutual diffusion take place, leading to the formation of a transitional mixing region between the two phases, as illustrated in Figure 4. By establishing the momentum and continuity equations for each phase in the two-phase flow, the differences in the physical properties of the polymer solutions result in varying momentum exchanges between the two phases. In the high-speed rotating nozzle, the observed slip between the two polymer solutions is essentially the interfacial slip between polymer solution I and polymer solution II. This study focuses on the slip behavior between these two distinct phases, where polymer solution I is defined as phase *i* and polymer solution II as phase *j*. The continuity equations for phase *i* and phase *j* are expressed as follows:

(2) $$\frac{\partial }{\partial t}\left(\alpha_{i}\rho_{i}\right)+\nabla \cdot \left(\alpha_{i}\rho_{i}\vec{v_{i}}\right)=0$$

(3) $$\frac{\partial }{\partial t}\left(\alpha_{j}\rho_{j}\right)+\nabla \cdot \left(\alpha_{j}\rho_{j}\vec{v_{j}}\right)=0$$

*α_i_* and *α_j_* represent the volume fractions of phases *i* and *j*, respectively. *ρ_i_* and *ρ_j_* denote the densities of phase *i* and *j*, while $\vec{v_{i}}$ and $\vec{v_{j}}$ correspond to their respective velocity vectors. The momentum conservation equation for phases *i* and *j* is expressed as follows:

(4) $$\frac{\partial }{\partial t}\left(\alpha_{i}\rho_{i}\vec{v_{i}}\right)+\nabla \cdot \left(\alpha_{i}\rho_{i}\vec{v_{i}}\vec{v_{i}}\right)=-\alpha_{i}\nabla i+\nabla \cdot \bar{\bar{\tau_{i}}}+\alpha_{i}\rho_{i}\vec{F_{i}}+\sum_{i=1}^{n}\vec{R_{ij}}$$

(5) $$\frac{\partial }{\partial t}\left(\alpha_{j}\rho_{j}\vec{v_{j}}\right)+\nabla \cdot \left(\alpha_{j}\rho_{j}\vec{v_{j}}\vec{v_{j}}\right)=-\alpha_{j}\nabla j+\nabla \cdot \bar{\bar{\tau_{j}}}+\alpha_{j}\rho_{j}\vec{F_{j}}+\sum_{j=1}^{n}\vec{R_{ji}}$$

$\overset{̿}{\tau_{i}}$ is the pressure strain tensor of phase *i*, $\vec{F_{i}}$ is the external volume force of phase *i*, and $\vec{R_{ij}}$ is the interaction force between the two phases.

**Figure 5:**
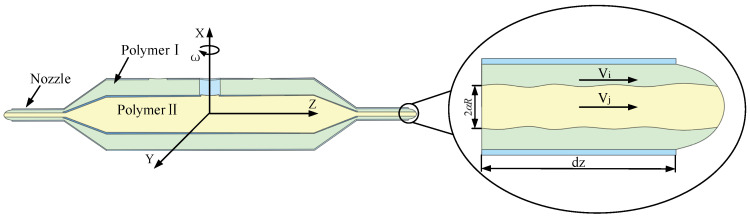
Schematic diagram of two-phase slip.

When two polymer solutions with different properties flow at velocities *V_i_* and *V_j_*, the concentration gradient between them induces a change in static pressure along the nozzle wall. This pressure variation generates a net force *f_ij_* in the flow direction, assuming no volume force acts in this direction. This net force influences the formation of two-phase flow waves within the polymer solution, as illustrated in Figure 5. The net force induced by the concentration gradient *∂α*/*∂z* is given by:

(6) $$f_{ij}=\left[\frac{\partial f_{ij}}{\partial \left(\frac{\partial \alpha }{\partial z}\right)}\right]\frac{\partial \alpha }{\partial z}$$

(7) $$f_{ij}=f_{\nabla \alpha }\left(\frac{\partial \alpha }{\partial z}\right)$$

*α* represents the volumetric density. The velocity of the two-phase flow wave in the polymer solution is given by:

(8) $$C={\left[\frac{-\left(v_{i}-v_{j}\right)}{\frac{\alpha }{\rho_{2}}+\frac{1-\alpha }{\rho_{1}}}-f_{\nabla \alpha }\right]}^{\frac{1}{2}}{\left(\frac{\rho_{1}}{1-\alpha }+\frac{\rho_{2}}{\alpha }\right)}^{-\frac{1}{2}}$$

When the liquid–liquid slip velocity satisfies the following equation, the polymer solution flow in the micro-triangle at the nozzle outlet stabilizes:

(9) $$v_{i}-v_{j}={\left[-f_{\nabla \alpha }\left(\frac{\alpha }{\rho_{2}}+\frac{1-\alpha }{\rho_{1}}\right)\right]}^{\frac{1}{2}}$$

Therefore, the liquid–liquid slip velocity is a critical factor determining the stable flow of the polymer solution in the micro-triangle at the nozzle exit, which is essential for forming high-quality composite fibers. Adjusting the concentrations of the two polymer solutions allows for the regulation of liquid–liquid slip velocity in the two-phase flow, enabling precise control over the internal composition of the composite fiber.

## 3. Simulation and Analysis of Micro-Triangle Slip Phenomenon

**Figure 6:**
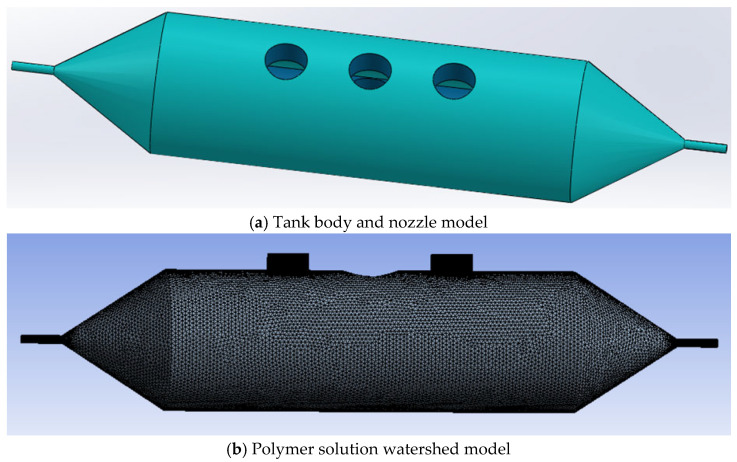
Rotary-force spinning tank and nozzle model.

The structure of the nozzle and tank used in rotary-force spinning is shown in Figure 6a. Two polymer solutions are injected through the feed hole at the top of the tank, which rotates at high speed under motor drive, forcing the solutions to flow rapidly toward the nozzle. As they exit the nozzle, the solutions undergo stretching and solvent evaporation in air, ultimately forming core–shell composite fibers. Therefore, the jet motion model of the polymer solutions primarily focuses on the velocity distribution within the nozzle.

The flow-field mesh division of the tank and nozzle during rotary-force spinning is shown in Figure 6b. In constructing the simulation model, most parameters were kept consistent with the actual spinning device. The key structural parameters include: tank length of 150 mm; inner and outer nozzle diameters of 0.6 mm and 2 mm; inlet diameter of 10 mm; and nozzle length of 10 mm. In the meshing software, ANSYS 2020 R2 software, the polymer solution domain was discretized using unstructured meshes with a maximum size of 1 mm. The inlet and outlet boundary layers were defined with thicknesses of 0.2 mm and 0.1 mm, respectively, with a total of three layers.

To ensure computational accuracy, a systematic grid refinement strategy was applied for grid independence verification. Using the base mesh with a maximum size of 1 mm as the reference, two refined meshes of 0.7 mm and 0.5 mm were generated at a refinement ratio of 1.5. All meshes maintained identical boundary-layer settings. As the deviation between the medium and finest grids was less than 1% and no significant difference in flow patterns was observed, it was confirmed that the 1 mm mesh provides a sufficiently grid-independent solution while effectively reducing computational cost.

After meshing the polymer solution basin and defining the boundary conditions, Fluent software 2020 R2 was employed to simulate the flow of two polymer solutions within the nozzle using a multiphase flow model. To investigate the effect of solution concentration on liquid–liquid slip velocity, HEC solutions (4 and 5 wt.%) and PEO solutions (4, 5, and 6 wt.%) prepared with deionized water were selected. Rheological properties of these solutions were measured using a rheometer and fitted to the power-law model (*τ* = *k·γ^n^*) to obtain the consistency index (*k*) and flow behavior index (*n*). These parameters were incorporated into Fluent for simulation, as summarized in Table 1.

**Figure 7:**
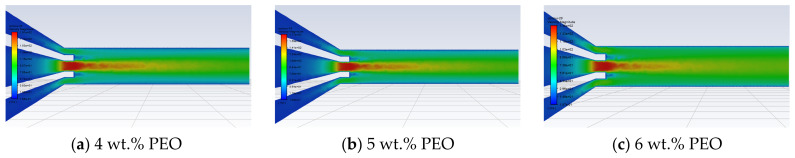
Clouds of velocity distribution of different PEO solution concentrations.

In addition, a 6 wt.% PEO solution and a 4 wt.% HEC solution were employed to simulate the polymer solution dynamics in rotary-force spinning under a nozzle diameter of 2 mm and motor speeds of 900, 1200, 1500, and 1800 rpm. The PEO solution was introduced through the central feeding hole at the top of the tank, while the HEC solution was supplied via the two side feeding holes. Figure 7 presents the velocity distribution cloud maps of 5 wt.% HEC solution combined with 4, 5, and 6 wt.% PEO solutions as they flow from the nozzle inlet to the outlet under conditions of a 1800 rpm motor speed and a 2 mm nozzle diameter.

**Figure 8:**
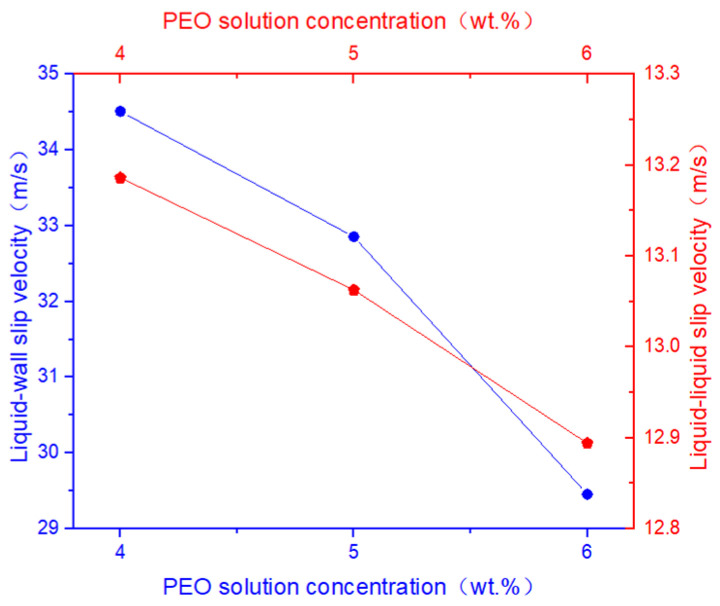
The liquid–liquid slip velocity and liquid–wall slip velocity at different PEO solution concentrations.

As shown in Figure 8, with increasing concentration of the PEO polymer solution, both the liquid–liquid slip velocity and the liquid–wall slip velocity at the nozzle outlet gradually decrease under the same rotational speed. The analysis indicates that higher PEO concentrations lead to a less pronounced liquid–liquid slip phenomenon within the micro-triangle. Simultaneously, the interaction between the two polymer solutions becomes more stable, which contributes to the formation of a more stable jet during the spinning process.

**Figure 9:**
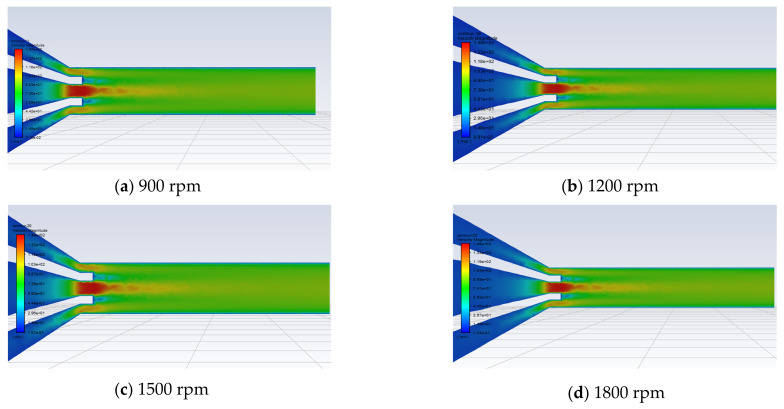
Clouds of velocity distribution of different rotational speeds.

**Figure 10:**
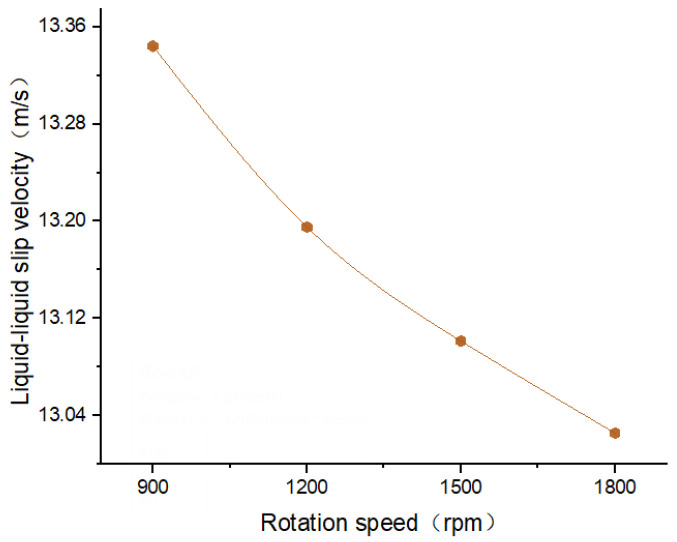
The Liquid–liquid slip velocity at different rotational speeds.

As illustrated in Figure 9 (cross-sectional velocity distribution cloud diagrams of 6 wt.% PEO solution and 4 wt.% HEC solution at different rotational speeds) and Figure 10 (liquid–liquid sliding velocity diagrams between the two polymer solutions at the nozzle outlet under different rotational speeds), the stability of the flow of the two polymer solutions within the nozzle improves with increasing motor rotational speed. As the rotational speed increases, the flow velocity distribution becomes more uniform and concentrated. Concurrently, the liquid–liquid slip velocity between the two polymer solutions decreases, indicating a more stable interaction at the interface.

## 4. Experiment on the Preparation of Composite Fiber Membranes by Rotational-Force Spinning Technology

In this rotational-force spinning experiment, polyoxyethylene (PEO, Mv = 1,000,000) and hydroxyethyl cellulose (HEC, 2% solution viscosity: 25–150 mPa·s at 25 °C) were selected as raw materials primarily due to their advantageous rheological and film-forming properties. PEO possesses relatively high viscosity, which enhances the rheological behavior of the spinning solution, ensuring jet stability during the spinning process and promoting the formation of uniform fibers. In contrast, HEC exhibits excellent film-forming ability and, when blended with other polymer materials, significantly improves the strength, toughness, and functional performance of the resulting fibers. Together, these two polymers play complementary roles in the fabrication of composite fibers.

**Figure 11:**
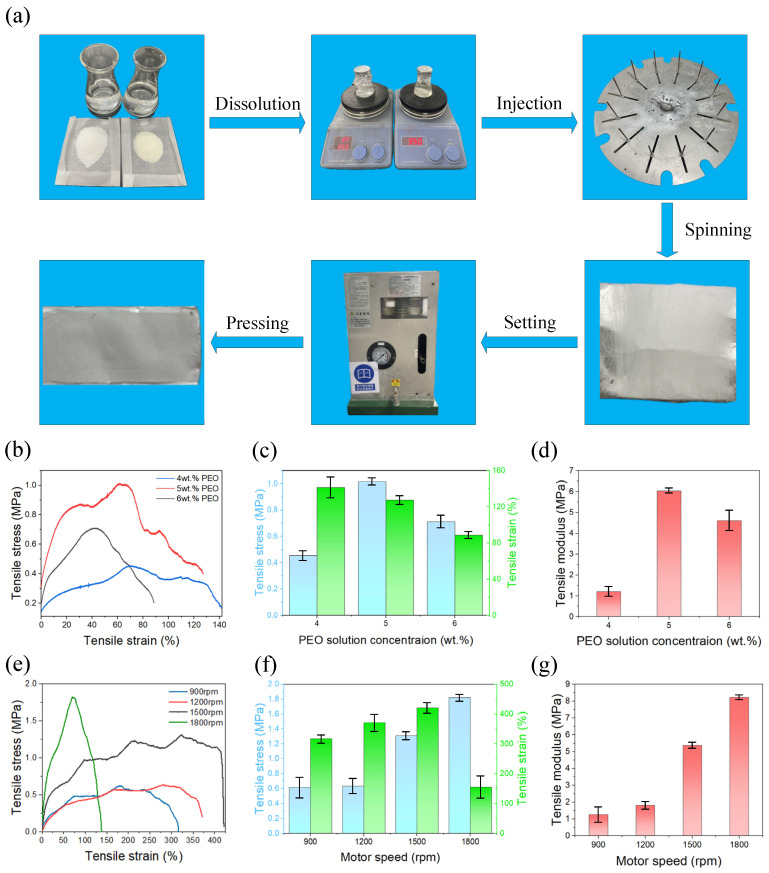
(**a**) Schematic illustration of the composite fiber membrane fabrication process. (**b**–**d**) Stress–strain curves, average stress–strain curves, and Young’s modulus of membranes prepared with different PEO concentrations. (**e**–**g**) Stress–strain curves, average stress–strain curves, and Young’s modulus of membranes prepared at different motor speeds.

In this experiment, as illustrated in Figure 11a, PEO and HEC powders were weighed separately and dissolved in deionized water. The solutions were stirred magnetically at 350 rpm for approximately 8 h at room temperature until homogeneous, yielding PEO solutions with concentrations of 4, 5, and 6 wt.% and HEC solutions with concentrations of 4 and 5 wt.%. The PEO solution was then introduced into the central feeding hole at the top of the tank using a syringe, while the HEC solution was fed into the two side holes. Rotary-force spinning was subsequently performed to fabricate PEO/HEC composite fibers. Finally, the collected fibers were pressed at 28 °C and 3 MPa for 25 min to obtain composite fiber films with a thickness of 0.1 mm and dimensions of 30 mm × 20 mm.

### 4.1. Mechanical Analysis of Peo/Hec Composite Fiber Membranes

Figure 11b–g present the stress–strain curves and mechanical properties of PEO/HEC composite fiber membranes, tested on an Instron 5943 universal testing machine (Norwood, MA, USA) at a tensile rate of 1 mm/min. Each sample was measured in triplicate, and the results are summarized in Table 2.

Under a motor speed of 1800 rpm and a nozzle diameter of 2 mm, composite fiber membranes prepared with 4, 5, and 6 wt.% PEO solutions (combined with 5 wt.% HEC solution) exhibited average tensile strengths of 0.45, 1.02, and 0.71 MPa; average tensile strains of 127.27%, 141.62%, and 88.60%; and Young’s moduli of 1.21, 6.04, and 4.61 MPa, respectively. These results indicate that both tensile strength and Young’s modulus first increased and then decreased with rising PEO concentration.

Furthermore, membranes fabricated using 6 wt.% PEO and 4 wt.% HEC solutions at motor speeds of 900, 1200, 1500, and 1800 rpm (nozzle diameter: 2 mm) displayed average tensile strengths of 0.62, 0.64, 1.31, and 1.82 MPa; average tensile strains of 316.89%, 371.18%, 421.40%, and 155.68%; and Young’s moduli of 1.27, 1.82, 5.37, and 8.22 MPa, respectively. These findings demonstrate that increasing motor speed markedly enhances the mechanical performance of the composite fiber membranes.

Overall, these results underscore the importance of optimizing both PEO concentration and motor speed to improve the microstructure, uniformity, and density of PEO/HEC composite fiber membranes.

### 4.2. Thermal Stability Analysis of Peo/Hec Composite Fiber Membranes

**Figure 12:**
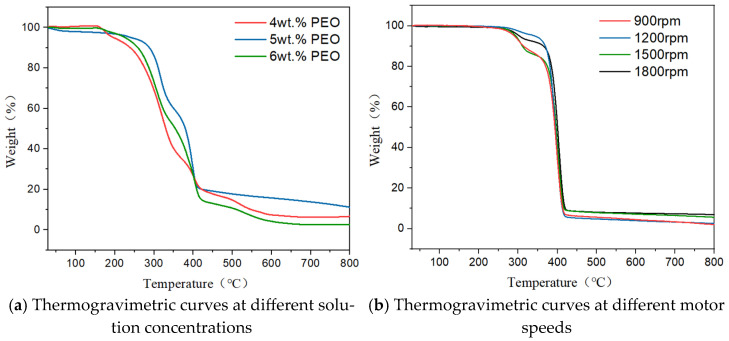
The TG curves of PEO/HEC composite fiber membranes under different PEO solution concentrations and motor speeds.

The thermal decomposition behavior of PEO/HEC composite fiber membranes was evaluated at a heating rate of 10 °C/min over a temperature range of 30–800 °C. As shown in Figure 12a,b, the membranes exhibited three main stages of weight loss.

In the first stage (30–200 °C for PEO/HEC membranes and up to 275 °C for certain samples), a mass loss of 3% was observed, primarily due to the evaporation of absorbed moisture. In the second stage (250–400 °C), the TG curve showed a sharp decline, corresponding to the thermal decomposition of the hydroxyethyl cellulose backbone and degradation of polyethylene oxide chains. At this stage, the mass loss reached approximately 80%. In the third stage (above 500 °C), the residue further carbonized, resulting in a total mass loss exceeding 80%.

Comparative TGA results demonstrated that, under a nozzle diameter of 2 mm, samples P5-H5-1800 and P6-H4-1800 exhibited the lowest decomposition rates among all tested conditions. This suggests that increasing both motor speed and PEO concentration enhances hydrogen bonding interactions between PEO and HEC molecules, thereby improving the thermal stability of the composite fiber membranes.

### 4.3. The Influence of Polymer Solution Concentration on the Morphology of Composite Fibers

**Figure 13:**
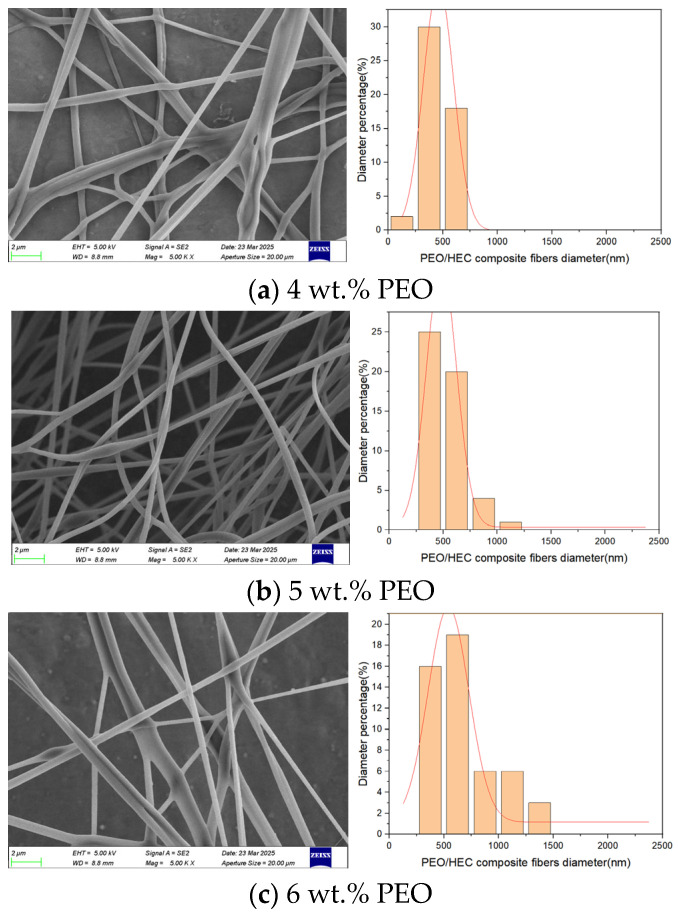
PEO/HEC composite fibers at different PEO solution concentrations.

To investigate the influence of polymer solution concentration on the morphology of composite fibers in composite fiber membranes, 4, 5, and 6 wt.% PEO solutions and a 5 wt.% HEC solution were used in this experiment. Three control variable experiments were conducted at a motor speed of 1800 rpm and a nozzle diameter of 2 mm. Based on the simulation results in Section 3, the liquid–liquid slip velocity in the micro-triangle changes with variations in polymer solution concentration. Adjusting the polymer solution concentration directly affects the tensile motion of the composite jet, thereby influencing the morphology of the composite fibers. Figure 13 presents SEM images showing the morphology of PEO/HEC composite fibers at different concentrations. At 4 wt.% PEO solution, the composite fibers’ surface is rough with numerous beads, and the diameter distribution is uneven. This results from the low-viscosity polymer solution causing unstable flow in the micro-triangle, leading to incomplete fiber stretching and the ejection of droplets. As the PEO solution concentration increases to 5 wt.%, the average diameter of the composite fibers increases, and the number of beads on the surface decreases. When the PEO solution concentration was further increased to 6 wt.%, the average diameter continued to increase, but the surface quality of the composite fibers deteriorated, and the diameter distribution became more dispersed.

The experimental results demonstrate that gradually increasing the concentration of the PEO polymer solution not only increases the average diameter of the composite fibers but also enhances the stability of the solution flow in the micro-triangle. However, excessively low concentrations hinder fiber formation, whereas excessively high concentrations may adversely affect the surface quality of the fibers.

### 4.4. The Influence of Rotational Speed on the Morphology of Composite Fibers

**Figure 14:**
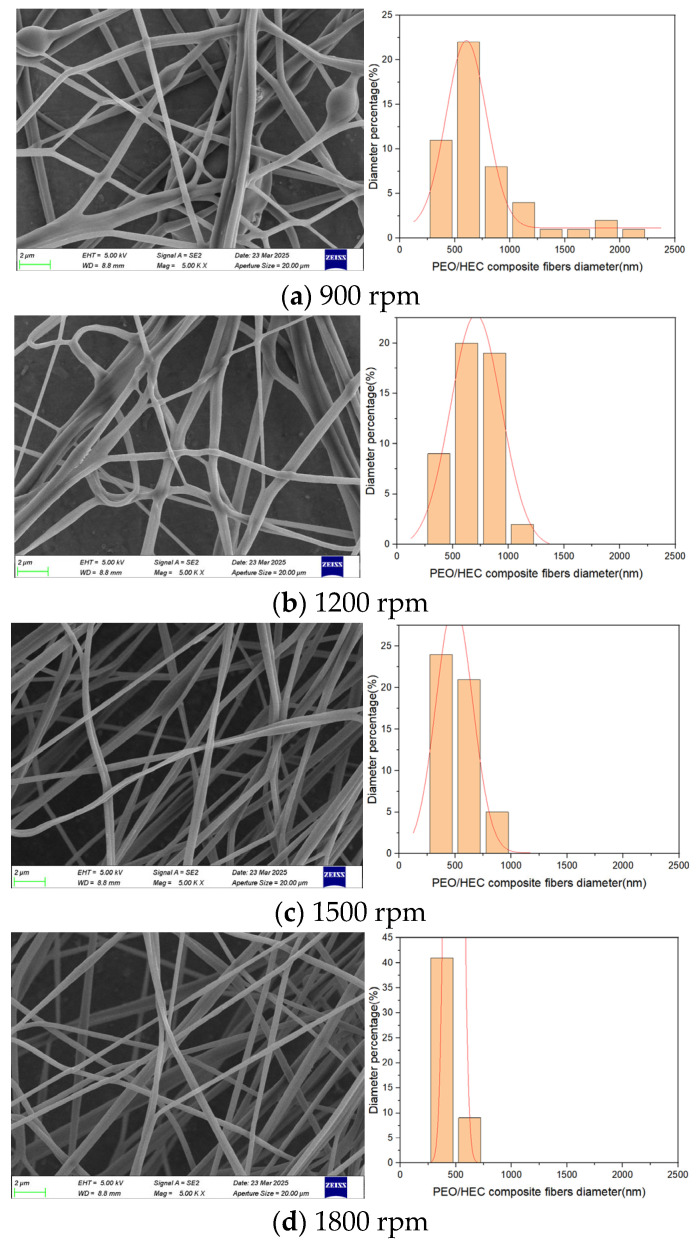
PEO/HEC composite fibers at different rotational speeds.

A rotational-force spinning device was employed. A 6 wt.% PEO solution and a 4 wt.% HEC solution were added to the tank, and a 2 mm nozzle was selected. Composite fiber membranes were fabricated at rotational speeds of 900, 1200, 1500, and 1800 rpm. Figure 14 displays SEM images of the composite fibers produced at rotational speeds ranging from 900 to 1800 rpm, along with their diameter distributions. At a rotational speed of 900 rpm, the diameters of the collected composite fibers were highly uneven, with a relatively large average diameter. Additionally, a small amount of droplet ejection was observed during the experiment. This phenomenon is attributed to the insufficient centrifugal force generated at lower rotational speeds, leading to expansion of the conical droplet at the nozzle outlet and a weak jet stretching effect in the air, ultimately resulting in larger fiber diameters. As the rotational speed increased from 1200 to 1800 rpm, SEM images revealed that the average fiber diameter decreased, the distribution became more uniform, and the surface appeared smoother. However, when the rotational speed reached 2100 rpm, complete fibers could no longer be collected on the receiving column.

The experimental results indicate that as the motor speed increases, the liquid–liquid slip behavior between the two polymer solutions becomes more stable, enabling the jet to achieve a steady stretching state. Consequently, the average diameter of the composite fibers gradually decreases, and their surface morphology becomes smoother and more uniform. However, when the rotational speed exceeds a certain threshold, fiber alignment deteriorates, leading to disordered deposition and reduced collection efficiency.

## 5. Conclusions

This paper presents a comprehensive investigation of a core–shell rotational-force spinning device and the fiber formation process occurring within the micro-triangle. A parametric model was developed to analyze the flow behavior and slip mechanism of polymer solutions, with particular emphasis on the influence of polymer concentration on liquid–liquid slip velocity. Numerical simulations conducted using Fluent revealed the flow field contours and velocity distributions inside the nozzle, showing that higher PEO concentrations and increased motor speeds reduce the liquid–liquid slip velocity, thereby enhancing flow stability within the micro-triangle. Experimental validation was performed using PEO and HEC as raw materials. The effects of motor speed and PEO concentration on the mechanical properties, thermal stability, fiber morphology, and diameter distribution of composite fiber membranes were systematically evaluated through mechanical testing, SEM, and TG analyses. The results confirmed that adjusting motor speed and PEO concentration effectively regulates liquid–liquid slip behavior, enabling precise control over fiber diameter and its distribution, while simultaneously improving mechanical strength, surface morphology, and thermal stability. Notably, under conditions of 1800 rpm and a 5 wt.% PEO concentration, the liquid–liquid slip reached its most stable state, significantly enhancing fiber quality. However, the lack of in situ techniques such as high-speed photography limits the full elucidation of the microscopic jet-forming mechanism in the micro-triangle. The “parameter–slipping–performance” framework established here offers a basis for future work, which will focus on using high-speed photography to capture interfacial evolution and, once the mechanism is clarified, to enable targeted, mechanism-driven optimization for practical application.

## Figures and Tables

**Table 1:** Rheological coefficients of two polymer solutions.

	4 wt.%HEC	5 wt.%HEC	4 wt.%PEO	5 wt.% PEO	6 wt.% PEO
k	6.800	17.839	1.750	4.096	11.066
n	0.637	0.568	0.729	0.661	0.571

**Table 2:** The tensile strength, tensile strain and Young’s modulus of the composite fiber membrane under different PEO polymer solution concentrations and motor speeds.

Sample	Tensile Strength (MPa)	Tensile Strain (%)	Young’s Modulus (MPa)
P4-H5-1800	0.45 ± 0.05	141.62 ± 2.4	1.21 ± 0.5
P5-H5-1800	1.02 ± 0.03	127.278 ± 1.18	6.04 ± 0.24
P6-H5-1800	0.71 ± 0.07	88.60 ± 0.88	4.61 ± 1.21
P6-H4-900	0.62 ± 0.11	316.89 ± 1.05	1.27 ± 2.13
P6-H4-1200	0.64 ± 0.09	371.18 ± 2.17	1.82 ± 1.05
P6-H4-1500	1.31 ± 0.04	421.40 ± 1.51	5.37 ± 0.73
P6-H4-1800	1.82 ± 0.03	155.68 ± 3.04	8.22 ± 0.42

## Data Availability

Due to the nature of this research, there were no participants in this study who needed to agree for their data to be shared publicly, so supporting data is not available.
